# Low Acrylamide Flatbreads Prepared from Colored Rice Flours and Relationship to Asparagine and Proximate Content of Flours and Flatbreads

**DOI:** 10.3390/foods10122909

**Published:** 2021-11-24

**Authors:** Xueqi Li, Talwinder Kahlon, Selina C. Wang, Mendel Friedman

**Affiliations:** 1 Olive Center, University of California, Davis, CA 95616, USA; spsli@ucdavis.edu; 2 Healthy Processed Foods Research, Western Regional Research Center, Agricultural Research Service, United States Department of Agriculture, Albany, CA 94710, USA; talwinder.kahlon@usda.gov; 3 Department of Food Science and Technology, University of California, Davis, CA 95616, USA

**Keywords:** pigmented rice flours, bioactive compounds, flatbreads, acrylamide, asparagine proximate analysis, food safety, human health

## Abstract

Acrylamide is a potentially toxic compound present in many plant-based foods, such as coffee, breads, and potato fries, which is reported to have carcinogenic, neurotoxic, and antifertility properties in vivo, suggesting the need to keep the acrylamide content of widely consumed food as low as possible. As pigmented rice contains bioactive phenolic and flavonoid compounds, the objective of this study was to potentially enhance the beneficial properties of flatbreads by evaluating the acrylamide content and proximate composition of 12 novel flatbreads prepared from the following commercial pigmented rice seeds: Black Japonica, Chinese Black, French Camargue, Himalayan Red, Long Grain Brown, Purple Sticky, Short Grain Brown, Wehani, Wild, Indian Brown Basmati, Organic Brown Jasmine, and Organic Jade Pearl. Although acrylamide levels ranged from 4.9 µg/kg in Long Grain Brown to 50.8 µg/kg in Chinese Black, the absolute values were all low (though statistically significantly differences existed among varieties). Acrylamide content did not correlate with its precursor asparagine. The variations in protein, carbohydrate, fat, ash, dry matter, and water content determined by proximate analysis, and the reported health benefits of colored rice cultivars used to prepare the flatbreads, might also be useful for relating composition to nutritional qualities and health properties, facilitating their use as nutritional and health-promoting functional foods.

## 1. Introduction

The heat-induced reactions of amino groups of amino acids, peptides, and proteins with the carbonyl groups of reducing sugars, such as glucose, result in the concurrent formation of so-called Maillard browning products and acrylamide [1]. Numerous worldwide studies suggest that baking, cooking, and frying of plant foods at temperatures above 120 °C induce the formation of the potentially toxic compound acrylamide during the course of Maillard-type browning reactions.

A systematic review and dose-response meta-analysis by Adani et al. [2] of the association between estimated dietary intake of acrylamide and risk of female cancer suggests that high acrylamide intake was associated linearly with increased risks of ovarian and endometrial cancer, and with the exception of premenopausal women, much less with breast cancer risk. On the basis of the observed mutagenicity of acrylamide and by the presence of the in vivo oxidation product glycidamide in mouse embryo fibroblasts, Hölzl-Armstrong et al. [3] suggested that glycidamide might be involved in the development of breast, ovarian, and lung cancers. On the basis of the results on acrylamide-induced neurotoxicity of cerebral organoids, Bu et al. [4] suggested that the risk of transplacental exposure of the fetus to acrylamide should be evaluated and that pregnant mothers should limit their exposure to the neurotoxin. Rayburn and Friedman [5] and Williams et al. [6] reported that L-cysteine, N-acetyl-L-cysteine, and reduced glutathione protected frog embryos ex vivo against acrylamide-induced mortality and organ malformations (teratogenicity). The results of a study by Ivanski et al. [7] on the effect of acrylamide on spermatogenesis in rats suggest that acrylamide consumption may impair their reproductive capacity and that it might also be involved in the observed increase in human infertility prevalence associated with male reproductive disorders. The cited studies imply that acrylamide might cause or contribute to human cancer, neurotoxicity, and infertility.

To minimize the reported adverse effects, it is important to keep the acrylamide content of the diet as low as possible. As part of this effort, we previously devised improved thermal processing conditions to reduce acrylamide formation in canned black ripe olives [8], prepared a set of gluten-free, low-acrylamide commercial and experimental flatbreads [9], and determined the effects of adding fruit and vegetable peels and mushroom powders to gluten-free experimental flatbreads on their acrylamide contents [10]. We also reported in this journal on low acrylamide flatbreads prepared from colored corn and other flours [11].

The main objective of the present study is to determine the acrylamide contents and proximate compositions of 12 experimental flatbreads prepared from pigmented black, brown, purple, pink, and red rice flours, and to determine possible relationships between these components and the asparagine contents of flours and flatbreads. Published studies on the health-promoting properties of the colored seeds and flours—most likely associated with their types of bioactive phenolic compounds—flavonoids, etc.; and protein, carbohydrate, and fat levels—are also mentioned to help consumers further in finding low-acrylamide, gluten-free, high protein, low-fat flatbreads.

## 2. Materials and Methods

### 2.1. Samples

The following rice seeds were purchased online from Purcell Mountain Farms, Moyie Springs, ID, USA (https://purcellmountainfarms.com/collections/rice, accessed on 20 November 2021): Black Japonica, Chinese Black, French Camargue, Himalayan Red, Long Grain Brown, Purple Sticky, Short Grain Brown, Wehani, and Wild. The following rice seeds were obtained from a local store (Costco, Richmond, CA, USA): Indian Brown Basmati, Organic Brown Jasmine, and Organic Jade Pearl.

### 2.2. Chemicals and Reagents

Optima LC/MS grade solvents of methanol, acetonitrile, formic acid, hexane, and water; and Carrez reagents I and II, were all purchased from Fisher Scientific (Waltham, MA, USA). Basix nylon filters (0.45 µm pore size) and disposable syringes were also purchased from Fisher Scientific. Analytical standards of acrylamide (≥99% purity) and acrylamide-d3 solution (500 mg/L in acetonitrile) were obtained from Millipore Sigma (Burlington, MA, USA).

### 2.3. Flatbread Preparation Process

Rice seeds were ground with a tabletop flour mill Blendtec Kitchen Mill Model 91 on a medium setting (Blendtec Inc., Orem, UT, USA). Flatbread dough was prepared by adding 48–54 mL water to 46–52 g of ingredients, as shown in Table 1. Several combinations of flatbread ingredients were cooked and tasted, and the final compositions of experimental flatbreads were decided based on consensus of laboratory personnel regarding the best taste. Flatbread ingredients were thoroughly mixed by hand with a large spoon in a large bowl. Water was then added until the dough began forming a ball. The dough was kneaded until it became smooth and elastic, placed in a 200 × 100 No. 3180 Pyrex bowl, covered with polyvinyl film (Polyvinyl Films, Sutton, MA, USA), and held at room temperature for 30 min. Each portion of dough (50 g) was placed on parchment paper (nonstick, oven-safe up to 216 °C) and pressed to a thickness of 1–1.5 mm, to a roughly 17 cm circle, in a 20 cm Alpine Cuisine flatbread Press (Aramco Imports, Inc., Commerce, CA, USA). Flatbreads were cooked between upper and lower hot irons of the flatbread maker for 2 min (1 min each side) at 165–195 °C on parchment paper in a 1000 Watt CucinaPro Flatbread Maker (SCS Direct, Inc., Trumbull, CT, USA). Flatbreads weighed ~32 g before drying and ~20 g after drying, indicating that cooked flatbreads on average contained 62.5% dry matter and 37.5% water.

### 2.4. Flatbread Proximate Composition Analysis

For proximate and acrylamide analysis, cooked flatbreads were chopped for 30 s in a Cuisinart coffee grinder Model DCG-20N (Cuisinart East Windsor, NJ, USA). Chopped flatbreads were then dried at 103 °C for 3 h. Complete dryness was confirmed with an additional 1 h of drying. Dried flatbreads were ground to fine powders using a coffee grinder (Cuisinart Model DCG-20N E, Windsor NJ, USA). Ground flatbreads were analyzed for nitrogen by combustion using AOAC method 990.03 with a Leco FP628 analyzer (Leco Corporation, St Joseph, MI, USA); for crude fat by Soxhlet extraction with petroleum ether using AOAC method 27.006; for ash using AOAC method 923.03; and for moisture using AOAC method 935.29 [12].

### 2.5. Analysis of Acrylamide Contents of Flatbreads

Acrylamide in flatbread samples was extracted and analyzed as previously described with minor modifications [9]. To start, 0.5 mL of acrylamide-d3 internal standard (400 µg/L) was added to 2.00 ± 0.01 g of flatbread powder and extracted with 19.5 mL methanol:water (80:20, *v/v*) using a stir plate (Southern Labware, Inc., Cumming, GA, USA) at 300 rpm for 20 min under ambient conditions. Subsequent centrifugation (8000 rpm, 5 min) was conducted on a Sorvall Legend X1 benchtop centrifuge (Thermo Scientific, Waltham, MA, USA) to collect the supernatant. The supernatant was mixed with 10 mL hexane and centrifuged (4000 rpm, 5 min) again to remove nonpolar fractions. An aliquot (0.1 mL) of Carrez reagents I and II was then added to further eliminate extract turbidity and emulsions. The cleaned supernatant was centrifuged (8000 rpm, 5 min) one more time, and 10 mL of the extract was evaporated to dryness at 35 °C on a Buchi Rotovap R-300 (New Castle, DE, USA). The sample was reconstituted in 1 mL Optima LC/MS grade water and filtered through nylon filters (0.45 µm) prior to HPLC injection.

An isocratic chromatographic separation was conducted on an Agilent C18 Eclipse Plus column (5 µm, 4.6 mm × 250 mm, Santa Clara, CA, USA) using a Waters Alliance 2695 separation module (Milford, MA, USA). Mobile phase A was 0.1% formic acid in water, and mobile phase B was formic acid (0.1%) in methanol:acetonitrile (50:50, *v/v*). The column was kept at 25 °C during analysis. The injection volume was 10 µL and the flow rate was 0.4 mL/min for a total of 12 min. The retention times of acrylamide and acrylamide-d3 were 9.01 and 8.92 min, respectively. Sample detection was performed on a Waters Quattro Micro API Mass Spectrometer using multiple reaction monitoring (MRM) in the positive electrospray ionization mode. The source temperature was 120 °C and the desolvation temperature was 400 °C. The capillary voltage was set to 2.75 kV, and the cone voltage was 20 V. Desolvation gas and cone gas flow rates were 600 L/h. and 25 L/h, respectively. Transitions for acrylamide and acrylamide-d3 were monitored at m/z (mass-to-charge ratio) 72 → m/z 54.8 and m/z 75 → m/z 57.9, respectively, with dwell times of 500 msec. A six-point calibration curve of concentration versus the peak area ratio of acrylamide to acrylamide-d3 was used for quantification (R^2^ = 0.9998). Accuracy, precision, and sensitivity of the analytical method for acrylamide were calculated and are elaborated in Results.

### 2.6. Analysis of Free Asparagine in Rice Flours and Flatbreads by Ion-Exchange Chromatography

This section was provided by Dr. Joshua Z. Yu, Amino Acid Analysis Laboratory, University of California, Davis. The finely ground sample (1.00 g) was measured into a 15 mL glass tube to which was added sulfosalicylic acid (3%, 10 mL). The tube was covered with parafilm, and the sample was evenly dispersed in the solution by vigorous shaking and kept overnight in refrigerator at 4 °C. The sample was further homogenized the following morning by vigorous shaking. The slurry (1 mL) was then poured into a 1.5 mL Eppendorf centrifuge tube (Hamburg, Germany) and centrifuged for 15 min. The supernatant solution was then loaded on to an HPLC Biochrom 30+ amino acid analyzer (Cambridge, UK) with an ion exchange column for amino acid separation and post column ninhydrin color derivatization for quantitation. The data represent the average concentrations with standard deviations (SD) from two independent extraction analyses using both rice flours and flatbreads [13].

### 2.7. Statistical Analysis

Statistical analysis of colored rice ingredients, flatbread proximate analysis, and flatbread acrylamide values were analyzed with Minitab software (version 14.12.0, Minitab Inc., State College, PA, USA) using basic statistics for mean ± standard deviation (SD) and one-way analysis of variance using Tuckey’s test. Multiple range test was used to determine significant differences among means and (*p* ≤ 0.05) was considered the criterion of significance. The Pearson product-moment correlation was determined with the aid of SigmaPlot 11 (San Jose, CA, USA).

## 3. Results and Discussion

### 3.1. Accuracy, Precision, and Sensitivity of the Analytical Method for Acrylamide

Each type of flatbread was prepared in triplicate, and the acrylamide content of each replicate was determined. One white corn flatbread replicate was randomly selected for routine analytical method validation. The recovery of acrylamide was calculated by spiking this white corn flatbread replicate with a known amount of acrylamide standard solution (50 µg/kg). A recovery rate of 90% was achieved. Intra-day and inter-day precision analyses were carried out using the same white corn flatbread replicate. Intra-day relative standard deviation (RSD) was calculated by running this replicate three times on the same day, whereas inter-day RSD was calculated by running this replicate once per day over three consecutive days, yielding values of 1.2% and 4.5%, respectively. Lastly, the limit of detection and the limit of quantification of the analytical method were determined as 3 and 10 times that of the signal-to-noise ratio, at 2.1 and 7.1 µg/kg, respectively. The protein, fat, carbohydrate, and ash contents of the samples did not seem to affect the extractions analysis for acrylamide.

### 3.2. Acrylamide Content of Flatbreads

**Figure 1:**
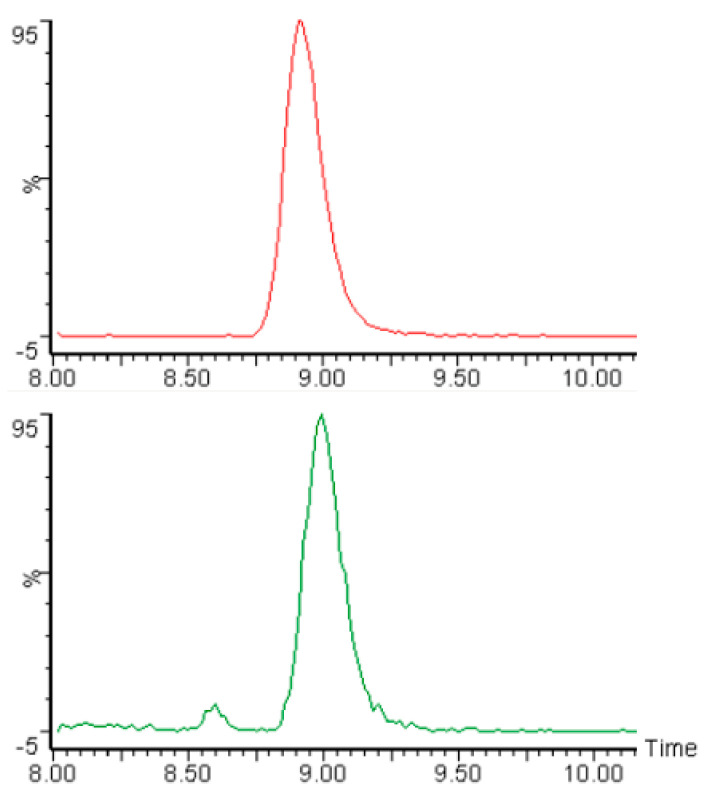
HPLC-MS/MS chromatograms of acrylamide-d3 (**top**) and acrylamide (**bottom**) in Black Japonica flatbread (50.5 ± 6.1 µg/kg). Y-axis: intensity; x-axis: retention time (min).

**Figure 2:**
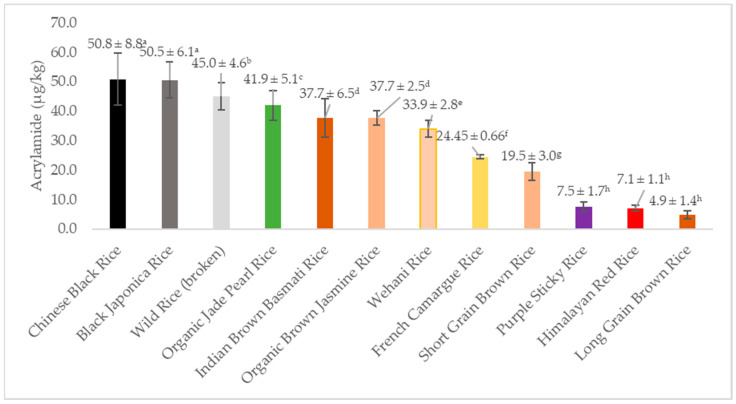
Trends in acrylamide content (µg/kg) of 12 flatbreads prepared from pigmented rice flours. Values shown are averages of three independent determinations with standard deviations (*n* = 3). Values with different superscript letters differ significantly (*p* ≤ 0.05).

Figure 1 shows an example of HPLC-MS/MS chromatograms of the internal standard (acrylamide-d3) and of acrylamide in Black Japonica flatbread. The average acrylamide values for the 12 flatbreads from three independent determinations with SD are shown in Table 2. They ranged from 4.9 µg/kg (Long Grain Brown) to 50.8 µg/kg (Chinese Black), which is a 13.7-fold variation from the lowest value to the highest value. The data also show that the acrylamide contents of the Black Japonica and Chinese Black flatbreads were similar (50.5 and 50.8 µg/kg, respectively), as were those of Wehani, Indian Brown Basmati, and Organic Brown Jasmine, with values of 33.9, 37.7, and 37.7 µg/kg, respectively. Noteworthy also are the very low values of Long Grain Brown, Himalayan Red, and Purple Sticky—4.9, 7.1, and 7.5 µg/kg, respectively. The colored bar graphs with standard deviations in Figure 2 visually illustrate the observed trends and the 13.7-fold variation in the acrylamide levels of the experimental flatbreads.

The results of the present study on flatbread acrylamide content can be compared with the results of an analysis by Esposito et al. [14] on the acrylamide content of 200 commercial bakery products sold by retail outlets in Italy. These authors reported that the acrylamide contents of breads and related bakery products baked under conditions which were likely different than we used for the flatbreads, ranged from 31 to 454 µg/kg and for sweets from 204 to 400 µg/kg. High-acrylamide results (in µg/kg) from cereal products reported by other investigators include (a) biscuits, up to 697 µg/kg [15]; cookies, up to 370 µg/kg [16,17]; (c) crackers, up to 300 µg/kg [18]; and (d) toasted bread, up to 200 µg/kg [19,20]. By contrast, whole wheat pita had a low level of 24 µg/kg [21], similar to those observed in the present study for the rice-based flatbreads.

On the basis of this comparison, it seems that the acrylamide levels in the flatbreads determined here are at the lower end of the scale of those of commercial bakery products, suggesting that the gluten-free, low-acrylamide flatbreads might not adversely affect the health of consumers. Moreover, the wide range in acrylamide contents of commercial cereal products suggests that consumers might also benefit if the acrylamide contents of breads are listed on the labels of such commercial breads. The highly sensitive, rapid assays for acrylamide used in the present study might be applicable to large-scale studies on the acrylamide contents of baked cereal products.

It is also worth noting that Bråthen and Knutsen [22] reported a positive relationship between the acrylamide contents of flatbreads and dry matter; that their colors varied from light brown when baked at low temperatures to almost black when baked at high temperature for a long time; and that the amount of acrylamide in a bread crust increased with the time and temperature of baking. We found that statistical Pearson correlations between acrylamide and proximate analysis were not significant, with the possible exception of fat content, which had a negative correlation of −0.595. We have no explanation for this result.

### 3.3. Proximate Composition of Flatbreads—Relationship to Acrylamide Content

Table 3 shows the proximate compositions of the flatbreads (in percentages) prepared from 12 pigmented rice flours. Composition was divided into crude protein calculated from nitrogen by combustion using a Leco FP628 analyzer—the multiplication factor used was 5.95—crude fat, ash, carbohydrate, dry matter, and water content. All values are expressed on a dry matter (DM) basis. The protein content ranged from 3.037% (Short Grain Brown) to 4.836% (Wild), or 1.59-fold variation from lowest value to highest value. The corresponding variation in fat content from 6.12% (Wild) to 10.13% (Black Japonica and Purple Sticky) is a 1.66-fold variation; for ash (mineral) content from 1.391% (Jade Pearl) to 2.400% (Wehani) is a 1.72-fold variation; for total carbohydrate from 44.76% (Chinese Black) to 48.68% (Short Grain Brown) is a 1.09-fold variation; for dry matter from 56.6% (Chinese Black) to 63.87% (Black Japonica) is a 1.13-fold variation; and for moisture content from 36.13% (Black Japonica) to 43.4% (Chinese Black) is 1.20-fold apparent variation. Pearson correlational analysis between proximate and acrylamide composition was unremarkable. Only crude fat content and acrylamide level had any significant correlation (*p* < 0.05), and the association was weak (−0.595). Because it is not known to what extent, if any, the variations in the proximate compositions—especially in the protein, fat, and carbohydrate levels—of the flatbreads could affect protein nutritional quality, it would benefit protein nutrition and food security to determine nutritional quality using the protein efficiency ratio (PER) mouse or rat growth assays [23,24].

### 3.4. Acrylamide–Asparagine Relationships of Flatbreads

Because the free amino acid asparagine is reported to be a major precursor in the mechanism of heat-induced acrylamide formation, we also determined the free asparagine contents of the 12 flours and the corresponding flatbreads made from these flours. Table 4 shows the free asparagine contents determined by analysis of free amino acids by ion-exchange chromatography. The results show excellent reproducibility of asparagine levels from two separate extraction analyses, and identical asparagine levels in flours and flatbreads. The Pearson correlation of acrylamide versus asparagine in rice flour was 0.493 whereas the Pearson correlation of acrylamide versus asparagine in rice flatbreads was 0.486, indicating other variables had low correlations. Only a small fraction of available asparagine was used in the formation of acrylamide, as indicated by the following values. Table 2 shows that acrylamide content (in µg/kg) ranged from 4.9 (Long Grain Brown Rice) to 50.8 (Chinese Black Rice), or a 10.4-fold variation from lowest value to highest value. Table 4 shows the corresponding range for asparagine in flours of 93.0 mg/kg (Organic Jade Pearl Rice Flour) to 1179 mg/kg (Chinese Black Rice Flour), or a 12.7-fold variation from the lowest value to highest value. The nearly identical asparagine ratios for the two flatbreads prepared from these flours (94.5 and 1085 mg/kg) were about 11.5. As flatbreads retain high amounts of asparagine, increasing the baking conditions (196 °C, 2 min) is expected to result in increased acrylamide content.

To place the results of the present study in perspective, here we mention the following brief highlights from the literature to illustrate the complexity of asparagine–acrylamide relationships. Surdyk et al. [25] reported that added asparagine increased the acrylamide content of dry bread crust from 80 µg/kg up to 6000 µg/kg. Pedreschi et al. [26] found that frying of potato strips produced French fries with up to 2075 µg/kg of acrylamide, and that treatment of blanched strips with the an asparaginase solution resulted in 60% reduced acrylamide content compared with frying without the added asparagine-hydrolyzing enzyme. Two publications by Nguyen et al. [27,28] describe the effects of asparagine during the baking of biscuits. The results showed that the acrylamide concentration was highest in biscuits with the highest concentrations of asparagine and glucose, and that there was no clear correlation between asparagine content and the sum of glucose and fructose concentrations in the wheat flour used to prepare the biscuits. Nan et al. [29] evaluated the effect of asparagine on the kinetics of inhibition of acrylamide formation by glutathione and quercetin in a model Maillard system. The results indicate that the predominant inhibition by glutathione on acrylamide formation might be attributed to the competitive reaction between glutathione and asparagine for the utilization of glucose in the formation of acrylamide. Yıltırak et al. [30] evaluated the effects of sprouting and fermentation by yeast on free asparagine and reducing sugars on acrylamide formation during the heating of several cereal flours. They found that acrylamide and 3-hydroxymethylfurfural formation decreased after fermentation of sprouted whole meal, apparently because asparagine and sugars that participate in acrylamide formation were partly metabolized by the yeast. The cited observations and the report by Vivanti et al. [31] that the asparagine contents of potatoes sold at retail in Italy and the United States vary widely suggest that knowledge about acrylamide precursors can serve as a guide to plant breeders, processors, and consumers to develop and use potatoes, cereals, and other processed plant foods with low amounts of precursors.

Furthermore, we ran an ANOVA of repeated measures on asparagine content between the before and after baking groups and found this to be significant (*p* < 0.05), suggesting that baking statistically reduced the asparagine content overall. A second ANOVA on the percentage of asparagine reduction between samples was not significant, suggesting that the individual samples were not significantly different regarding reduction in asparagine content. Finally, a Pearson correlation between acrylamide and the percentage of asparagine reduction showed that asparagine reduction was not correlated with acrylamide content.

### 3.5. Health Benefits of Pigmented Rice and Its Components

As the low-acrylamide, gluten-free flatbreads prepared from pigmented rice flours contain biologically active health-promoting phenolics and anthocyanin compounds, here, we briefly mention select highlights from the literature on the compositions of reported bioactive compounds and their health benefits to provide insight into the expected benefits of the flatbreads derived from colored rice varieties that might serve as health-promoting functional foods.

Poulev et al. [32] reports that the purple rice flavone tricin, located in the bran (pericarp) part of purple rice, inhibits colon cancer in animals. Friedman [33] described the health-promoting potential of rice-derived hulls, brans, and bran oils in cells, animals, and humans in relation to composition. Ito and Lacerda [34] suggested that anthocyanins and phenolic compounds in black rice provide health benefits. Fracassetti et al. [35] found that cooking pigmented rice cultivars resulted in lower reductions in bioactive compounds than cooking by boiling, microwaving, or pressure cooker. Melini and Acquistucci [36] reported that cooking pigmented Thai and wild rice induced decreases in bioactive anthocyanins, carotenoids, and phenolic compounds. Chu et al. [37] developed an extraction procedure for wild rice to facilitate the determination of proanthocyanin content. A randomized trial on 19 human volunteers by Vitalini et al. [38] showed that consumption of two black rice bran cultivars significantly increases plasma levels of polyphenols and flavonoids, and antioxidative capacity. A prospective cohort study showed that multi-grain rice diets decreased the risk of breast cancer in Korean women (*n* = 93,306) by 33% as compared to a 35% higher risk for those consuming white rice diets [39]. Dong et al. [40] found that red yeast rice attenuates atherosclerosis in mice. Chen et al. [41] assessed the relationships of the compositions of pigmented rice bran extracts with inhibition of cancer cells. Kim et al. [42] found that feeding tumor-bearing mice the black rice bran component γ-oryzanol resulted in a significant reduction in colon tumor mass. Choi et al. [43] reported that feeding mice diets supplemented with 10% (*w/w*) black or brown rice bran for 2 weeks reduced the growth of transplanted tumors by 35% or 19%, respectively. Kim et al. [44] also showed that a fermented black rice bran formulation inhibits the growth of the foodborne pathogen *Salmonella enterica* in infected mice. Qi et al. [45] identified the beneficial effect of the flavonoid quercetin on Alzheimer’s disease biomarkers, and Khan et al. [46] found that quercetin also inhibits the fibril formation of amyloid-β proteins, counteracting inflammatory pathways associated with brain disorders and suggesting that the flavonoids present in colored rice [47] might ameliorate this neurodegenerative illness in humans. Finally, Kim et al. [48] reported that a fermented black rice bran formulation inhibited ethyl alcohol-induced hangovers in mice and rats. As the molecular mechanism of the in vivo hangover effect induced by alcohol-derived acetaldehyde (CH_3_-CH=O) described in Kim et al. [48] is similar to that of bioalkylation of essential proteins [49] and DNA [50] by acrylamide [(CH_2_=CH-C=O(NH_2_)], it is likely that the new functional anti-hangover food formulation can also inhibit the adverse in vivo effects of acrylamide.

These cited studies imply that the new flatbreads prepared from milled colored rice seeds and rich in phenolic and flavonoid compounds have the potential to serve as functional foods that might ameliorate the adverse effects of multiple diseases.

## 4. Conclusions

In conclusion, the objective of the present study is to determine the acrylamide contents and proximate compositions of 12 experimental flatbreads prepared from pigmented black, brown, purple, pink, and red rice flours, and to determine possible relationships between these components and the asparagine contents of flours and flatbreads. Due to the flatbreads’ low potentially toxic acrylamide levels ranging from 4.9 to 50.8 µg/kg, the experimental flatbreads might be safer to consume than traditionally-produced commercial flatbreads [9]. The proximate analysis data in Table 3 show that consumers will be able to select gluten-free flatbreads with high protein or low fat content. Moreover, the rice cultivars used to prepare the flatbreads have been reported to contain multiple biologically active, health-promoting compounds, and so the resulting flatbreads have the potential to ameliorate the adverse effects of several diseases, an aspect that merits further study. Low-acrylamide, gluten-free flatbreads with potential health benefits prepared from colored rice flours represent new functional foods that merit commercial production and marketing by food processors and bakeries as well as preparation and consumption in restaurants and homes.

## Figures and Tables

**Table 1:** Flatbread dough composition *.

Flatbread	Rice Flour (g)	Guar Gum (g)	Salt (g)	Olive Oil (mL)	Water (mL)
Black Japonica Rice	47.82	1.91	0.53	1.91	47.82
Chinese Black Rice	41.82	1.67	0.46	1.67	54.37
French Camargue Rice	47.82	1.91	0.53	1.91	47.82
Himalayan Red Rice	47.82	1.91	0.53	1.91	47.82
Long Grain Brown Rice	47.82	1.91	0.53	1.91	47.82
Purple Sticky Rice	46.71	1.87	0.51	1.87	49.04
Short Grain Brown Rice	47.82	1.91	0.53	1.91	47.82
Wehani Rice (red-brown)	46.71	1.87	0.51	1.87	49.04
Wild Rice (broken; black)	43.65	1.75	0.48	1.75	52.38
Indian Brown Basmati Rice	47.82	1.91	0.53	1.91	47.82
Organic Brown Jasmine Rice	47.82	1.91	0.53	1.91	47.82
Organic Jade Pearl Rice (green)	41.82	1.67	0.46	1.67	54.37

* Liquids were measured in mL and dry ingredients in g.

**Table 2:** Acrylamide contents of 12 flatbreads prepared from pigmented rice flours (*n* = 3).

Flour Type	Acrylamide * (µg/kg)
Black Japonica Rice	50.5 ± 6.1 ^a^
Chinese Black Rice	50.8 ± 8.8 ^a^
French Camargue Rice	24.45 ± 0.66 ^f^
Himalayan Red Rice	7.1 ± 1.1 ^h^
Long Grain Brown Rice **	4.9 ± 1.4 ^h^
Purple Sticky Rice	7.5 ± 1.7 ^h^
Short Grain Brown Rice	19.5 ± 3.0 ^g^
Wehani Rice	33.9 ± 2.8 ^e^
Wild Rice (broken)	45.0 ± 4.6 ^b^
Indian Brown Basmati Rice	37.7 ± 6.5 ^d^
Organic Brown Jasmine Rice	37.7 ± 2.5 ^d^
Organic Jade Pearl Rice	41.9 ± 5.1 ^c^

* Acrylamide values are mean ± SD; values with different superscript letters differ significantly (*p* ≤ 0.05); analyses were conducted in triplicate (*n* = 3). ** Long grain brown acrylamide value was below the limit of quantification (7.1 µg/kg), but the estimated value is shown.

**Table 3:** Proximate compositions of rice flatbreads prepared from pigmented rice flour on a dry matter basis *.

Flatbreads	Protein ** (N × 5.95) (g)	Crude Fat (g)	Ash (g)	Total Carbohydrates (g)	Dry Matter (DM) (g)	Water (mL)
Black Japonica Rice	4.512 ± 0.071 ^b^	10.13 ± 0.33 ^a^	2.355 ± 0.010 ^a^	47.73 ± 0.52 ^d^	63.87 ± 0.43	36.13 ± 0.43
Chinese Black Rice	3.107 ± 0.040 ^h^	7.55 ± 0.30 ^d^	2.048 ± 0.030 ^g^	44.76 ± 1.3 ^h^	56.6 ± 1.1	43.4 ± 1.1
French Camargue Rice	3.665 ± 0.050 ^f^	8.92 ± 0.42 ^b^	2.3260 ± 0.0067 ^b^	47.90 ± 0.62 ^c^	61.93 ± 0.68	38.06 ± 0.68
Himalayan Red Rice	3.637 ± 0.010 ^f^	9.78 ± 0.24 ^a^	1.9850 ± 0.0093 ^f^	48.06 ± 0.18 ^c^	62.73 ± 0.47	37.27 ± 0.47
Long Grain Brown Rice	3.685 ± 0.027 ^f^	9.70 ± 0.68 ^a^	2.297 ± 0.013 ^bc^	47.60 ± 0.24 ^d^	62.42 ± 0.50	37.58 ± 0.50
Purple Sticky Rice	4.027 ± 0.20 ^d^	10.13 ± 0.62 ^a^	2.2300 ± 0.0048 ^d^	46.81 ± 0.94 ^f^	62.36 ± 0.17	37.63 ± 0.17
Short Grain Brown Rice	3.037 ± 0.51 ^i^	9.09 ± 0.18 ^b^	2.233 ± 0.010 ^d^	48.68 ± 0.50 ^a^	62.199 ± 0.055	37.801 ± 0.055
Wehani Rice	3.818 ± 0.53 ^e^	9.30 ± 0.78 ^b^	2.400 ± 0.014 ^b^	46.08 ± 0.50 ^g^	60.6 ± 1.2	39.4 ± 1.2
Wild Rice (broken)	4.836 ± 0.72 ^a^	6.12 ± 0.64 ^f^	2.268 ± 0.019 ^e^	46.14 ± 0.28 ^g^	58.42 ± 0.32	41.58 ± 0.32
Indian Brown Basmati Rice	4.417 ± 0.84 ^c^	8.91 ± 0.48 ^bc^	2.164 ± 0.040 ^e^	47.42 ± 2.31 ^e^	62.1 ± 1.5	37.9 ± 1.5
Organic Brown Jasmine Rice	3.256 ± 0.11 ^g^	7.77 ± 0.56 ^d^	2.047 ± 0.010 ^f^	48.56 ± 1.3 ^a^	60.83 ± 0.62	39.16 ± 0.62
Organic Jade Pearl Rice	3.247 ± 0.069 ^g^	7.23 ± 0.54 ^de^	1.391 ± 0.029 ^h^	48.26 ± 1.4 ^b^	59.5 ± 1.7	40.5 ± 1.7

* Liquids were measured in mL and dry ingredients in g. ** Protein, crude fat, ash, and total carbohydrate values are mean ± SD, values with different superscript letters differ significantly (*p* ≤ 0.05); analyses were conducted in triplicate (*n* = 3).

**Table 4:** Individual and average (*n* = 2 ± SD) asparagine contents of rice flours and flatbreads (mg/kg).

	Flour Asparagine	Flatbread Asparagine
	507.0 ± 9.9	458.5 ± 6.4
Chinese Black Rice	1179 ± 32	1085 ± 17
French Camargue Rice	427 ± 15	377 ± 16
Himalayan Red Rice	218 ± 11	205.50 ± 0.71
Long Grain Brown Rice	146.5 ± 2.1	173.0 ± 8.5
Purple Sticky Rice	116.0 ± 8.5	120.0 ± 2.8
Short Grain Brown Rice	284.0 ± 2.8	251.5 ± 4.9
Wehani Rice	382 ± 13	304 ± 18
Wild Rice (broken)	130.5 ± 3.5	147.0 ± 2.8
Indian Brown Basmati Rice	405.0 ± 4.2	367.0 ± 4.2
Organic Brown Jasmine Rice	216.0 ± 2.8	211.50 ± 0.71
Organic Jade Pearl Rice	93.0 ± 1.4	94.5 ± 3.5

Pearson correlation of acrylamide versus asparagine in rice flour = 0.493; Pearson correlation of acrylamide versus asparagine in rice flatbreads = 0.486.

## Data Availability

Data is contained within the current article.
